# Both IIC and IID Components of Mannose Phosphotransferase System Are Involved in the Specific Recognition between Immunity Protein PedB and Bacteriocin-Receptor Complex

**DOI:** 10.1371/journal.pone.0164973

**Published:** 2016-10-24

**Authors:** Wanli Zhou, Guohong Wang, Chunmei Wang, Fazheng Ren, Yanling Hao

**Affiliations:** 1 The Innovation Centre of Food Nutrition and Human Health (Beijing), College of Food Science and Nutritional Engineering, China Agricultural University, Beijing, China; 2 Key Laboratory of Functional Dairy, Co-constructed by Ministry of Education and Beijing Municipality, Beijing, China; University Medical Center Utrecht, NETHERLANDS

## Abstract

Upon exposure to exogenous pediocin-like bacteriocins, immunity proteins specifically bind to the target receptor of the mannose phosphotransferase system components (man-PTS IIC and IID), therefore preventing bacterial cell death. However, the specific recognition of immunity proteins and its associated target receptors remains poorly understood. In this study, we constructed hybrid receptors to identify the domains of IIC and/or IID recognized by the immunity protein PedB, which confers immunity to pediocin PA-1. Using *Lactobacillus plantarum* man-PTS EII mutant W903, the IICD components of four pediocin PA-1-sensitive strains (*L*. *plantarum* WQ0815, *Leuconostoc mesenteroides* 05–43, *Lactobacillus salivarius* REN and *Lactobacillus acidophilus* 05–172) were respectively co-expressed with the immunity protein PedB. Well-diffusions assays showed that only the complex formed by LpIICD from *L*. *plantarum* WQ0815 with pediocin PA-1 could be recognized by PedB. In addition, a two-step PCR approach was used to construct hybrid receptors by combining LpIIC or LpIID recognized by PedB with the other three heterologous IID or IIC compounds unrecognized by PedB, respectively. The results showed that all six hybrid receptors were recognized by pediocin PA-1. However, when IIC or IID of *L*. *plantarum* WQ0815 was replaced with any corresponding IIC or IID component from *L*. *mesenteroides* 05–43, *L*. *salivarius* REN and *L*. *acidophilus* 05–172, all the hybrid receptors could not be recognized by PedB. Taken altogether, we concluded that both IIC and IID components of the mannose phosphotransferase system play an important role in the specific recognition between the bacteriocin-receptor complex and the immunity protein PedB.

## Introduction

Bacteriocins, a class of antimicrobial peptides that are produced by bacteria, can inhibit growth or kill bacteria, while having diverse spectra of activity, i.e. from targeting only strains that are closely related to the producer strains or to a broader range of bacteria [1]. Pediocin-like bacteriocins are typically characterized by the presence of a conserved YGNGVXCXXXXCXV peptide motif within their N-terminal domains and also by a strong inhibitory effect on *Listeria* [2]. Pediocin-like bacteriocins contain two domains: a cationic N-terminal domain and a more hydrophobic C-terminal domain determining the target cell specificity [3]. The strong inhibitory effect of pediocin-like bacteriocins on food pathogens, such as *Listeria*, suggests multiple applications as antimicrobials in food and feed industry [4].

Generally, genes encoding pediocin-like bacteriocins are co-transcribed with or in the vicinity of a gene encoding a cognate immunity protein that protects the bacteriocin-producer from being killed by their own bacteriocins [5]. Although immunity proteins show remarkably high degrees of specificity to the bacteriocin they recognize, some of these immunity proteins may also provide immunity to other pediocin-like bacteriocins [6]. The C-terminal domains of pediocin-like immunity proteins specifically recognize the C-terminal hairpin domain of bacteriocins, resulting in immunity to bacteriocins [7, 8]. Sprules and colleagues suggested that the hydrophobic pocket within the C-terminal domain of the immunity proteins may be attracting the immunity proteins to the surface of the cell membrane [9].

The mannose phosphotransferase system (man-PTS), which is a major sugar uptake system in Gram-positive and Gram-negative bacteria, consists of EI, Hpr and EII enzymes [10]. EII is composed of four distinct components: IIA, IIB, IIC and IID. IIA and IIB are normally represented by a single cytoplasmic protein (IIAB) and the membrane-located proteins IIC and IID together form a functional receptor for pediocin-like bacteriocins [11]. A recent study has shown that an extracellular loop of the IIC protein determined specificity for class IIa bacteriocins [12]. Immunity proteins form a strong complex with the bacteriocins and receptor, therefore protecting bacteriocin-producer cells [11]. The detailed mechanism of recognition of the membrane-located components IIC and IID by the immunity protein still remains unclear. In the present study, we demonstrated that both IIC and IID components play an important role in the formation of the bacteriocin-receptor-immunity protein complex using a series of hybrid receptors.

## Materials and Methods

### Bacterial strains, plasmids and growth conditions

The list of bacterial strains and plasmids used in this study are shown in Table 1. *Lactobacillus* spp. and *Leuconostoc mesenteroides* 05–43 were grown under anaerobic conditions at 37°C in de Man-Rogosa-Sharpe (MRS). *Lactococcus lactis* NZ9000 was propagated at 30°C in M17 broth supplemented with 0.5% w/v D-glucose. *Escherichia coli* were cultured in Lysogeny broth (LB) medium with shaking at 37°C. Pediocin PA-1 was produced by *Lactobacillus plantarum* strain LB-B1, which was originally and previously isolated from a traditionally fermented dairy product [13]. When required, relevant antibiotics (Sigma-Aldrich) were added at the following final concentrations: 50 μg/ml of kanamycin (Km), 10 μg/ml chloramphenicol (Cm) or 500 μg/ml of erythromycin (Em) for *E*. *coli* and 5 μg/ml Em or 10 μg/ml Cm for *L*. *plantarum*, respectively.

**Table 1:** Bacterial strains and plasmids used in this study.

Strains or plasmids	Characteristics	Reference or source
**Bacterial strains**		
*E*. *coli* EC1000	RepA^+^ MC1000, kanamycin resistance, carrying a single copy of the pWV01 *repA* gene; host for pORI28-based plasmids	[14]
*E*. *coli* ECHEA	*E*. *coli* EC1000 containing plasmid pORIHEA	This study
*E*. *coli* MC1061	Plasmid-free strain; *araD* 139, *Δ*(*ara-leu*)7697, *Δ*(*lac*)X74, *galU*^-^, *galK*^-^, *hsdR*^-^, *rpsL*	[15]
*L*. *lactis* NZ9000	Plasmid-free strain; MG1363 *pepN*: *nisRK*	[1]
*L*. *plantarum* LB-B1	Pediocin PA-1 producing strain (isolated from fermented dairy product)	[13]
*L*. *plantarum* WQ0815	Pediocin PA-1-sensitive strain (isolated from Sichuan Pickle)	Laboratory collection
*L*. *mesenteroides* 05–43	Pediocin PA-1-sensitive strain(isolated from fermented dairy product)	Laboratory collection
*L*. *acidophilus* 05–172	Pediocin PA-1-sensitive strain (isolated from fermented dairy product)	Laboratory collection
*L*. *salivarius* REN	Pediocin PA-1-sensitive strain (isolated from the feces of a healthy centenarian)	[16]
*L*. *plantarum* W903	man-PTS EII mutant of *L*. *plantarum* WQ0815; Em^r^	This study
**Plasmids**		
pTRK669	Ori (pWV01), Cm^r^, RepA^+^	[17]
pORI28	Em^r^, ori (pWV01), replicates only with RepA provided *in trans*	[14]
pORIHEA	2.3 Kb; pORI28 with 725-bp internal man-PTS *IIAB* fragment of *L*. *plantarum*	This study
pNZ8148	Gene expression vector, P_nisA_, Cm^r^	[1]
pNZ8300	5.4 Kb; pNZ8148 with nisin regulatory genes *nisRK*	This study

### DNA manipulation techniques

Mini-prep plasmid isolations from both *E*. *coli* and *L*. *plantarum* were performed using the E.Z.N.A^TM^ Plasmid Mini Kit Ι according to the manufacturer’s instructions (OMEGA Bio-tek Inc., Doraville, GA, USA). Total DNA from *Lactobacillus* spp., *Leuconostoc* spp., and *Lactococcus* spp., were isolated using the genomic DNA extraction kit (Tiangen, Beijing, CN). Lysis of the bacterial cells was operated by adding lysozyme to TES buffer (50 mM Tris-Cl, 1 mM EDTA, 25% sucrose; pH 8.0) to a final concentration of 30 mg/ml, and the mixtures were incubated at 37°C for 1 h [18]. Plasmids were electroporated into *E*. *coli* and *L*. *plantarum* as described previously [19, 20]. PCR products were amplified by using *Ex Taq* polymerase (Takara, Dalian, CN). Restriction endonuclease digestions were performed according to the supplier’s instructions (Takara, Dalian, CN). DNA ligations were performed using the DNA Ligation kit (Tiangen, Beijing, CN). DNA sequences were determined with the Bigdye Terminator cycle sequencing kit (Sangon, Beijing, CN).

### Construction of WQ0815 man-PTS EII mutant

The site-directed integration system described previously by Russell and Klaenhammer [17] was used to inactivate man-PTS EII in the genome of *L*. *plantarum* strain WQ0815. Genes encoding IIAB, IIC and IID components of EII are located within the same gene cluster, so the insertional inactivation of gene *IIAB* will also inactivate the downstream genes *IIC* and *IID*. A 725-bp internal region of the man-PTS *IIAB* gene of *L*. *plantarum* WQ0815 was chosen as a homologous sequence to construct WQ0815 man-PTS EII mutant. It was amplified using the forward primer HEAF (5’-CATGCCATGGCGGAATTTGCATATATATAAGTGAG-3’) and the reverse primer HEAR (5’-CGCGGATCCGTCCGCAGTTCGTCTTTAG-3’), which were designed according to the gene *IIAB* (Accession no. CP002222.1). Restriction sites used for subsequent clones are underlined: *Nco*I and *Bam*HI for the forward and reverse primers, respectively. These two sites were used to clone the amplified product into the integration plasmid pORI28 [14]. The recombinant plasmid, designated as pORIHEA, was transformed into *L*. *plantarum* WQ0815 containing pTRK669. Plasmid pTRK669 with chloramphenicol resistant (Cm^r^) gene is a temperature-sensitive helper plasmid that provides RepA in *trans* for the replication of pORI28. A temperature increase from 37°C to 42°C resulted in the integration of plasmid pORIHEA into the WQ0815 genome and also the loss of pTRK669 and its associated chloramphenicol resistance phenotype. Plasmid pORIHEA was integrated into *IIAB* gene region through homologous recombination in the chromosome of *L*. *plantarum* WQ0815. The resulting mutant (erythromycin resistant) was designated *L*. *plantarum* W903. Then, PCR with the forward primer EmrF (5’-TTTTAGCAAACCCGTATTCCAC-3’) and the reverse primer MptAR (5’-CCAAAATACCTTCCATACC-3’) was performed to confirm the integration of pORIHEA at the correct genome locus. The primer EmrF was designed according to the DNA sequence of erythromycin resistance (Em^r^) genes (Accession no. KM017875.1) and the primer MptAR was designed according to the DNA sequence of man-PTS gene (Accession no. CP002222.1).

### Selection of receptors and recognition by the immunity protein PedB

*L*. *plantarum* WQ0815, *L*. *mesenteroides* 05–43, *L*. *salivarius* REN and *L*. *acidophilus* 05–172 are sensitive to pediocin PA-1. The receptors from these four strains could be recognized by the pediocin PA-1. For co-expressing different genes of interest at the same time, we changed the sequence of the original multiple cloning sites of plasmid pNZ8148 by introducing other restriction sites. The modified multiple cloning sites contained *Nco*I, *Nde*I, *Sph*I, *Pst*I, *Sac*I, *Kpn*I, *Xho*I, *Eco*RI, *Spe*I, *Xba*I and *Hin*dIII sites. Then in order to induce expression of the genes of interest in W903, the *nisRK* genes from *L*. *lactis* NZ9000 were cloned into this plasmid using *Xba*I and *Hin*dIII restriction sites, creating a new plasmid pNZ8300. Next, *lpIICD* from *L*. *plantarum* WQ0815, *lmIICD* from *L*. *mesenteroides* 05–43 and *laIICD* from *L*. *acidophilus* 05–172 were respectively cloned into pNZ8300 between *Nco*I and *Xho*I sites to produce the recombinant plasmids, designated pNZlpCD, pNZlmCD and pNZlaCD, respectively. *Sph*I and *Xho*I sites were used to clone the *lsIICD* genes from *L*. *salivarius* REN into pNZ8300, resulting in the recombinant plasmid pNZlsCD. These vectors were then transformed into *L*. *plantarum* W903, the sensitivity of these four recombinant strains to pediocin PA-1 was determined by the well-diffusion assay. In order to further examine whether the expressed man-PTS EII components could serve as receptors recognized by the immunity protein PedB, the immunity protein gene *pedB* was inserted to the downstream of *lpIICD*, *lmIICD*, *lsIICD* and *laIICD* genes in pNZ8300, respectively. The *pedB* gene was obtained by PCR from the chromosomal DNA of *L*. *plantarum* LB-B1, using the primers Im-F (5’-CCGCTCGAGGGCATCAATAAAGGGGTG-3’) and Im-R (5’-TGCTCTAGACTATTGGCTAGGCCACGTATTG-3’), which were designed according to the gene *pedB* (Accession no. AJ242489.1). Restriction sites used for subsequent cloning are underlined: *Xho*I and *Xba*I for the forward and reverse primers, respectively. The digested PCR products were respectively inserted into pNZlpCD, pNZlmCD, pNZlsCD and pNZlaCD to produce the recombinant plasmids, designated pNZlpCD-B, pNZlmCD-B, pNZlsCD-B and pNZlaCD-B, respectively. Then all the recombinant plasmids were transformed into *L*. *plantarum* W903, the sensitivity of all recombinant strains to pediocin PA-1 was determined by the well-diffusion assay. All the primer sequences for amplifying genes encoding IIC and IID components are given in Table 2.

**Table 2:** PCR primers for amplifying genes *IIC* and *IID*.

Primer	DNA sequence^a^
lpcF	5’-CATGCCATGGGCATGAATTTGAACGTAATTC-3’; *Nco*I
lpdR	5’-CCGCTCGAGCCCACGAGTCCATCCTTT-3’; *Xho*I
lmcF	5’-CATGCCATGGCTATGTCTGTTATTGCGATAG-3’; *Nco*I
lmdR	5’-CCGCTCGAGGCCAGATTGACAAGCACT-3’; *Xho*I
lscF	5’-ACATGCATGCATGAGTACGATTCAAATT-3’; *Sph*I
lsdR	5’-CCGCTCGAGTTATAGTAAACCAATTAC-3’; *Xho*I
lacF	5’-CATGCCATGGGAATGAACGCTATACAAATG-3’; *Nco*I
ladR	5’-CCGCTCGAGTTAAAGAATGTGCCATACG-3’; *Xho*I
lpmcR	5’-CCATCATCTTGCCCCTTTCTGCAGCTAATACTTGTCGATAAT-3’
lpmdF	5’-ttatcgacaagtatTAGCTGCAGAAAGGGGCAAGATGATGG-3’
lmpcR	5’-CTCAGTTTGCCTCCTTCTCTGCAGCTAGTACTTGTTCAAAAT-3’
lmpdF	5’-ATTTTGAACAAGTACTAGCTGCAGAGAAGGAGGCAAACTGAG-3’
lpscR	5’-gtttttcccctcctaagCTAATACTTGTCGATAAT-3’
lpsdF	5’-ttatcgacaagtatTAGcttaggaggggaaaaac-3’
lspcR	5’-ctcagtttgcctccttctTTAGATGTCTTCCAAAAT-3’
lspdF	5’-attttggaagacatcTAAagaaggaggcaaactgag-3’
lpacR	5’-ctcctctacgcgttcattCTAATACTTGTCGATAAT-3’
lpadF	5’-ttatcgacaagtatTAGaatgaacgcgtagaggag-3’
lapcR	5’-CTCAGTTTGCCTCCTTCTttaataatcatcaatga-3’
lapdF	5’-tcattgatgattatTAAagaaggaggcaaactgag-3’

^a^Restriction site sequences are underlined.

### Construction of hybrid receptor

In order to determine the part(s) or domains of IIC and/or IID that are responsible for specific recognition by immunity protein PedB, we constructed hybrid receptors by combining LpIIC or LpIID recognized by PedB with the heterologous IID or IIC compounds unrecognized by PedB, respectively. In this study, six different combinations of genes of *lpIIC* with *lmIID*, *lmIIC* with *lpIID*, *lpIIC* with *lsIID*, *lsIIC* with *lpIID*, *lpIIC* with *laIID*, and *laIIC* with *lpIID* were generated using a two-step PCR approach [12]. In this procedure, two separate DNA fragments were amplified in the first step by using one outer primer and one inner primer for each fragment. Overlapped sequences were introduced using the inner primers and the two fragments were fused in a second PCR using the outer primers. Hybrid receptor genes were cloned into pNZ8300 between *Nco*I and *Xho*I sites, or *Sph*I and *Xho*I sites to produce the recombinant plasmids, designated pNZlpClmD, pNZlmClpD, pNZlpClsD, pNZlsClpD, pNZlpClaD and pNZlaClpD, respectively. Then the recombinant plasmids were transformed into *L*. *plantarum* W903. The sensitivity of the recombinant strains to pediocin PA-1 was determined by well-diffusion assay. Only hybrid receptors recognized by pediocin PA-1 were used in this study, because self-protection by the immunity protein only takes place after the formation of the bacteriocin receptor complex [11]. Furthermore, the PCR product of *pedB* gene was respectively cloned into pNZlpClmD, pNZlmClpD, pNZlpClsD, pNZlsClpD, pNZlpClaD and pNZlaClpD between *Xho*I and *Xba*I sites to produce the recombinant plasmids, designated pNZlpClmD-B, pNZlmClpD-B, pNZlpClsD-B, pNZlsClpD-B, pNZlpClaD-B and pNZlaClpD-B, respectively. These recombinant plasmids were also transformed into *L*. *plantarum* W903. The sensitivity of these six recombinant strains to pediocin PA-1 was determined by the well-diffusion assay. All recombinant plasmids were verified by DNA sequencing analyses. The primers and DNA templates used to construct hybrid receptors are detailed in Table 3.

**Table 3:** Outline of the cloning procedure.

Plasmids	Outer primer	Inner primer	Template
pNZlpCD	lpcF	-^a^	Chromosomal DNA of *L*. *plantarum* WQ0815
	lpdR		
pNZlmCD	lmcF	-^a^	Chromosomal DNA of *L*. *mesenteroides* 05–43
	lmdR		
pNZlsCD	lscF	-^a^	Chromosomal DNA of *L*. *salivarius* REN
	lsdR		
pNZlaCD	lacF	-^a^	Chromosomal DNA of *L*. *acidophilus* 05–172
	ladR		
pNZlpClmD	lpcF	lpmcR	pNZlpCD
	lmdR	lmpdF	pNZlmCD
pNZlmClpD	lmcF	lpmcR	pNZlmCD
	lpdR	lmpdF	pNZlpCD
pNZlpClsD	lpcF	lpscR	pNZlpCD
	lsdR	lspdF	pNZlsCD
pNZlsClpD	lscF	lpscR	pNZlsCD
	lpdR	lspdF	pNZlpCD
pNZlpClaD	lpcF	lpacR	pNZlpCD
	ladR	lapdF	pNZlaCD
pNZlaClpD	lacF	lpacR	pNZlaCD
	lpdR	lapdF	pNZlpCD

^a^No two-step PCR was necessary for this construct.

### Bacteriocin activity assays

Pediocin PA-1 was prepared using ammonium sulfate precipitation (70% saturation) from the culture supernatant of *L*. *plantarum* LB-B1. The precipitate was dissolved in a 10 mM phosphate buffer (pH 6.0) followed by filtration by a 0.2 μm pore size filter. Bacteriocin activity was expressed in units, and one bacteriocin unit (U) was defined as the amount of bacteriocin required to reduce the growth of the indicator strain *L*. *plantarum* WQ0815 by 50% under the tested conditions of the assay [21]. Bacteriocin activity was assayed by the well-diffusion method. When the recombinant strains were used as the indicator strains, the well-diffusion assay was modified by adding nisin (200 ng/ml) in the soft agar with 10^6^ CFU of the indicator strains [22]. For the bacteriocin activity assay, 128 U bacteriocin was added into each metal punch on the plates.

## Results

### Inactivation of man-PTS EII genes in *L*. *plantarum* WQ0815 by homologous recombination

**Fig 1:**
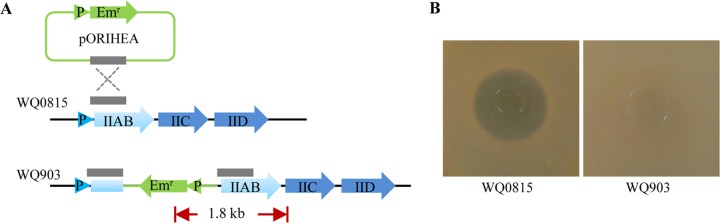
(A) Construction of the *L*. *plantarum* mutant strain W903. Genes are represented by arrows, promoters are indicated by triangles, and the internal fragment of *IIAB* is represented by a gray solid box. Chromosomal DNA is represented by black lines, plasmid DNA is represented by green lines, and the red arrow indicates the PCR products amplified using the forward primer EmrF and the reverse primer MptAR. (B) Sensitivity of *L*. *plantarum* WQ0815 (left) and *L*. *plantarum* W903 (right) to pediocin PA-1.

In order to eliminate the interference of the endogenous receptor, a *L*. *plantarum* WQ0815 man-PTS EII gene insertion mutant was constructed in this study. The mutant was constructed as shown in Fig 1A. To confirm the integration of pORIHEA at the correct genome locus, PCR was performed using the primers EmrF from sequence of Em^r^ gene and MptAR from man-PTS gene. The PCR result showed that an expected 1.8-kb fragment could be observed, when chromosomal DNA from the mutant W903 was used as template (S1 Fig). Sequence analysis showed that this PCR product contained the expected fragments of the Em^r^ gene and man-PTS IIAB gene. When chromosomal DNA from the wild-type strain WQ0815 was used as template in the PCR, no product was generated. This result indicated that the Em^r^ gene has been integrated into the chromosome of mutant W903 by homologous recombination.

Furthermore, the sensitivity of this mutant to pediocin PA-1 was tested by the well-diffusion assay. As shown in Fig 1B, an inhibition zone was observed on the plate of the wild-type strain WQ0815. However, the mutant W903 was resistant to pediocin PA-1, suggesting that receptor EII has been inactivated in this mutant. The PCR result, combined with the well-diffusion assay results, demonstrated that the single crossover homologous recombination event was successful.

### The immunity protein PedB recognizes the specific receptor

**Fig 2:**
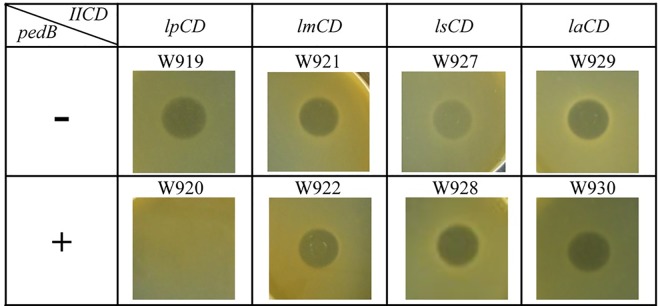
Sensitivity of *L*. *plantarum* W903 derivatives harboring gene *IICD* or *IICD* and *pedB* to pediocin PA-1.

In order to study recognition specificities between these four different receptors and the immunity protein PedB, all recombinant plasmids with the different receptors were first transformed into the host *L*. *plantarum* W903, the well-diffusion assay was employed to investigate the sensitivity of the recombinant strains to pediocin PA-1. The results showed that only the control strain W918 (W903 with pNZ8300) was still resistant to pediocin PA-1 (S2 Fig), whereas all the recombinant strains W919 with *lp*CD, W921 with *lmCD*, W927 with *lsCD* and W929 with *laCD* became sensitive to pediocin PA-1 (Fig 2), suggesting that LpIICD, LmIICD, LsIICD and LaIICD could be recognized by pediocin PA-1 in the host W903. Furthermore, the well-diffusion assays showed that only the recombinant strain W920 with *lpCD-B* was resistant to pediocin PA-1, while the recombinant strains W922 with *lmCD-B*, W928 with *lsCD-B* and W930 with *laCD-B* were still sensitive to pediocin PA-1 (Fig 2). The results indicated that the immunity protein PedB could only recognize the complex formed by protein IICD from *L*. *plantarum* WQ0815 with pediocin PA-1 and could not recognize the complex formed by proteins IICD from *L*. *mesenteroides* 05–43, *L*. *salivarius* REN or *L*. *acidophilus* 05–172 with pediocin PA-1.

### Both IIC and IID components play an important role in specific recognition by immunity protein PedB

**Fig 3:**
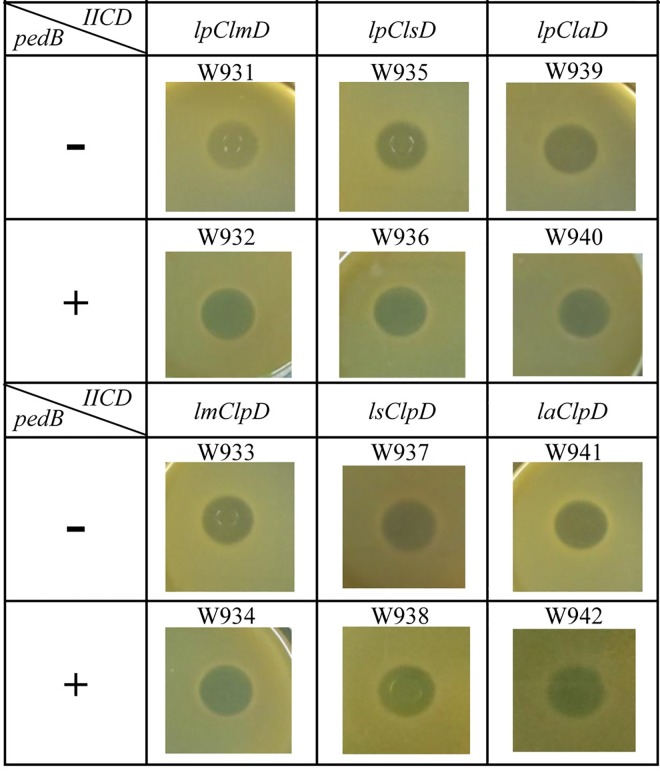
Sensitivity of *L*. *plantarum* W903 derivatives harboring hybrid *IIC/IID* or *IIC/IID* and *pedB* to pediocin PA-1.

The well-diffusion assays showed that all the recombinant strains W931, W933, W935, W937, W939 and W941 produced sensitivity to pediocin PA-1, suggesting that all six hybrid-type receptor genes were successfully expressed in W903 (Fig 3). These hybrid receptors form a complex with pediocin PA-1, allowing us to determine whether the immunity protein could provide immunity or not to pediocin PA-1. Well-diffusion assays showed that all the recombinant strains expressing the hybrid receptor genes and *pedB* gene in W932, W934, W936, W938, W940 and W942 still remained sensitive to pediocin PA-1, which indicated that IIC component of *L*. *plantarum* WQ0815 could not be replaced by IIC components of *L*. *mesenteroides* 05–43, *L*. *salivarius* REN or *L*. *acidophilus* 05–172 in the formation of the immunity protein-receptor-bacteriocin complex. The IID component of *L*. *plantarum* WQ0815 could also not be replaced with the other three kinds of corresponding IID components. These results demonstrated that both IIC and IID components play important roles in specific recognition by the immunity protein PedB. Taken altogether, we concluded that the immunity protein PedB directly interacts with the IIC protein, IID protein and pediocin PA-1 to form a complex, therefore preventing membrane permeabilization and cell death.

## Discussion

Pediocin-like bacteriocins have a strong inhibitory effect on *Listeria*, which illustrates their potential applications as antimicrobials in the food and feed industry. For the sensitive strains, both membrane-located proteins IIC and IID of man-PTS were confirmed to be necessary for the receptor function [11]. When heterologously expressing IIC or IID components, these may interact with the endogenous IID or IIC protein in *L*. *plantarum* and together form a hybrid receptor complex that resulted in sensitivity to pediocin-like bacteriocins [12]. In order to eliminate the interference of endogenous receptor, *L*. *plantarum* WQ0815 man-PTS EII gene insertion mutant was constructed by site-directed integration system.

In a previous work, the leucocin A immunity gene was heterologously expressed in *Enterococcus faecalis* and the resulting strain displayed immunity to enterocin A, pediocin PA-1 and leucocin A, whereas in *Lactobacillus sakei* and *Carnobacterium piscicola*, this gene could only provide immunity to leucocin A [23], indicating that strain-specific factors may influence the function of immunity proteins. In this study, we co-expressed immunity protein PedB with receptor IICD from *L*. *plantarum* WQ0815, *L*. *mesenteroides* 05–43, *L*. *salivarius* REN or *L*. *acidophilus* 05–172 in the host W903. Our results showed that only the receptor from *L*. *plantarum* WQ0815 could recognize the immunity protein, suggesting that the receptor contributed to strain-specific immune protein functionality. In order to investigate whether there are other host-specific factors involved in immunity, in our previous work, man-PTS IICD genes of *L*. *plantarum* WQ0815 and *L*. *mesenteroides* 05–43 were expressed in bacteriocin-resistant strain *L*. *lactis* NZ9000 using the NICE system. The well-diffusion assay indicated that man-PTS IICD of these two strains could be recognized by pediocin PA-1. The immunity protein was respectively co-expressed in *L*. *lactis* with man-PTS IICD of these two strains. The result showed that PedB could recognize the man-PTS IICD of *L*. *plantarum* WQ0815, but could not recognize these components of *L*. *mesenteroides* 05–43, suggesting that there are no other host-specific factors involved in the specific recognition between immunity protein and receptors [24].

**Fig 4:**
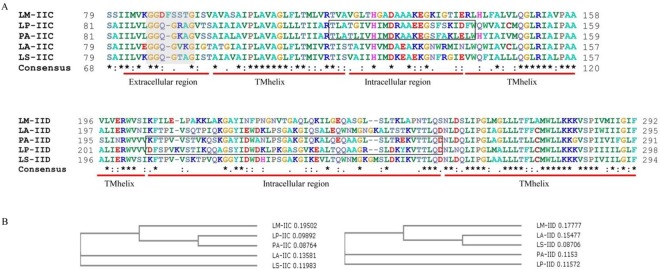
**Multiple sequence alignments (A) and phylogenetic clustering (B) of IIC and IID proteins from *L*. *plantarum*, *L*. *mesenteroides*, *L*. *salivarius*, *L*. *acidophilus* and *P*. *acidilactici*.** Transmembrane helix (TMhelix), extracellular and intracellular regions were determined by using TMHMM v. 2.0 software. An asterisk, two dots, and one dot indicated decreasing degrees of conservation. The conserved motifs GGQGxxG and GG[D/K]FxxxG in the extracellular loop region are indicated by a grey background. The residues from *L*. *plantarum* and *P*. *acidilactici* in the intracellular regions are indicated by boxes. Sequence alignments and phylogenetic trees were constructed by using MUSCLE v. 3.8.31 software with default settings (http://www.ebi.ac.uk/Tools/msa/muscle/) [27].

Sequence alignment of man-PTS IIC and IID proteins from *L*. *plantarum*, *L*. *mesenteroides*, *L*. *salivarius*, *L*. *acidophilus* and *Pediococcus acidilactici* has been carried out. Given that *P*. *acidilactici* is also a pediocin PA-1 producer, its IICD components can be recognized by immunity protein PedB. The amino acid sequence of PedB of *P*. *acidilactici* showed 100% identity to that of *L*. *plantarum* LB-B1, suggesting that the IICD from *P*. *acidilactici* can be recognized by PedB from *L*. *plantarum* LB-B1. Therefore, the IICD of *P*. *acidilactici* as a PedB-recognized receptor is also included in the sequence alignment. Comparative amino acid sequence analysis of IIC proteins showed that all five IIC homologs contain the conserved motif GGQGxxG or GG[D/K]FxxxG in their extracellular loop regions (Fig 4A), which has previously been reported to be critical for the interaction with class II bacteriocins [25].

Furthermore, multiple sequence alignments of IIC and IID proteins revealed two regions with higher heterogeneity than the rest of the sequence, corresponding to residues 120 to 140 in IIC and 210 to 262 in IID of *L*. *plantarum*, respectively. Transmembrane prediction using TMHMM [26] indicated that these two regions are both located on the intracellular side of the membrane (Fig 4A). Since immunity protein protects cells by forming a complex with IICD and bacteriocin, these intracellular loop regions might be involved in the specific interaction between immunity protein and IICD components. In addition, compared to IICD proteins of *L*. *mesenteroides*, *L*. *salivarius* and *L*. *acidophilus*, phylogenetic analysis suggested that IIC and IID proteins of *L*. *plantarum* are closer to the corresponding component of *P*. *acidilactici*. (Fig 4B). Only IICD from *P*. *acidilactici* and *L*. *plantarum* can be recognized by the immunity protein PedB, suggesting that specific residues located in these intracellular loop regions might serve as potential binding sites for PedB. Site-directed mutations will be carried out in future studies to further investigate the role of the residues in these regions in the interaction between receptor and immunity protein.

## Supporting Information

S1 Fig Agarose gel electrophoresis of PCR product obtained with the primers EmrF and MptAR. Lane M, a molecular weight marker. Lane 1, PCR product without template DNA; lane 2, PCR product with chromosomal DNA from WQ0815 as a template; lane 3, PCR product with chromosomal DNA from W903 as a template. The arrow indicates the 1.8-kb amplified product.
(TIF)

S2 Fig Sensitivity of *L*. *plantarum* W918 to pediocin PA-1. (TIF)
