# Supplementary figures and images for: Both IIC and IID Components of Mannose Phosphotransferase System Are Involved in the Specific Recognition between Immunity Protein PedB and Bacteriocin-Receptor Complex
Source: PLoS One. 2016 Oct 24;11(10):e0164973. doi: 10.1371/journal.pone.0164973 (PMC5077127; doi:10.1371/journal.pone.0164973)

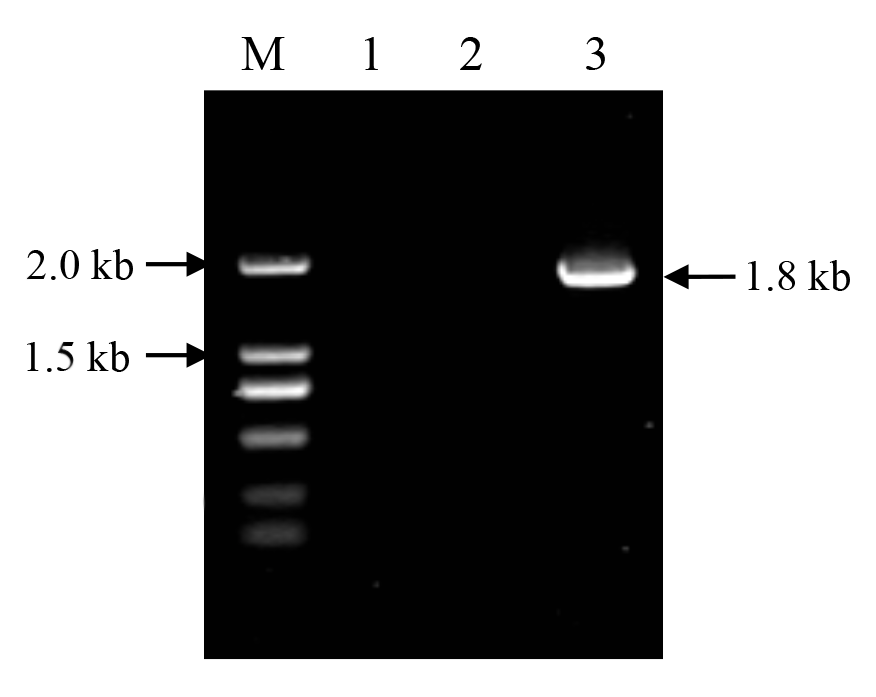

Supplement: S1 Fig — Lane M, a molecular weight marker. Lane 1, PCR product without template DNA; lane 2, PCR product with chromosomal DNA from WQ0815 as a template; lane 3, PCR product with chromosomal DNA from W903 as a template. The arrow indicates the 1.8-kb amplified product. (TIF) [file pone.0164973.s001.tif]

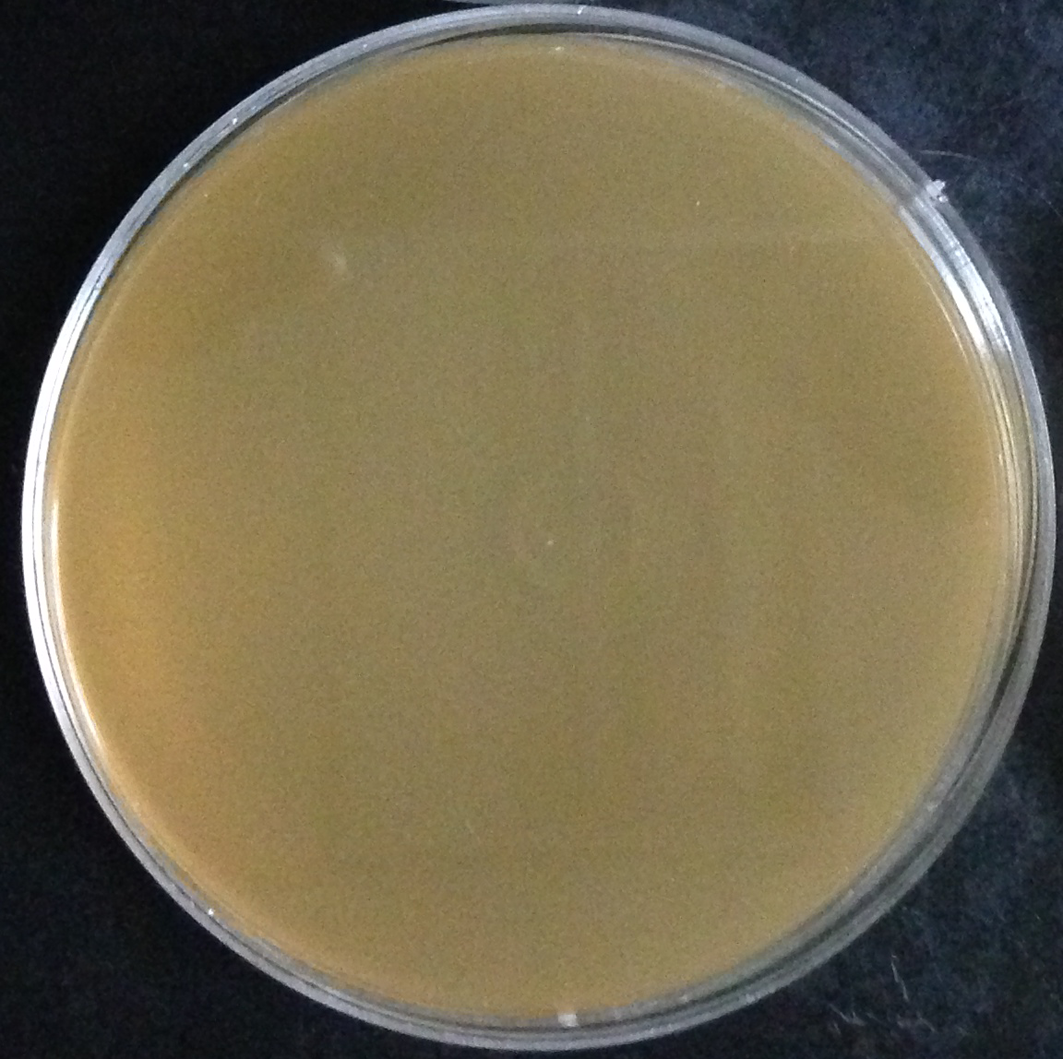

Supplement: S2 Fig — (TIF) [file pone.0164973.s002.tif]
